# Human Lung Tissue Transcriptome: Influence of Sex and Age

**DOI:** 10.1371/journal.pone.0167460

**Published:** 2016-11-30

**Authors:** Matteo Dugo, Chiara E. Cotroneo, Emilie Lavoie-Charland, Matteo Incarbone, Luigi Santambrogio, Lorenzo Rosso, Maarten van den Berge, David Nickle, Peter D. Paré, Yohan Bossé, Tommaso A. Dragani, Francesca Colombo

**Affiliations:** 1 Department of Experimental Oncology and Molecular Medicine, Fondazione IRCCS Istituto Nazionale dei Tumori, Milan, Italy; 2 Department of Predictive and Preventive Medicine, Fondazione IRCCS Istituto Nazionale dei Tumori, Milan, Italy; 3 Institut Universitaire de cardiologie et de pneumologie de Québec, Québec, Canada; 4 Department of Surgery, San Giuseppe Hospital–MultiMedica, Milan, Italy; 5 Fondazione IRCCS Ospedale Maggiore Policlinico, University of Milan, Milan, Italy; 6 University of Groningen, University Medical Center Groningen, Department of Pulmonology, Groningen Research Institute for Asthma and COPD (GRIAC), Groningen, The Netherlands; 7 Merck & Co. Inc., Rahway, NJ, United States of America; 8 University of British Columbia Center for Heart Lung Innovation and Institute for Heart and Lung Health, St. Paul’s Hospital, Vancouver, BC, Canada; 9 Respiratory Division, Department of Medicine, The University of British Columbia, Vancouver, BC, Canada; 10 Department of Molecular Medicine, Laval University, Québec, Canada; Defense Threat Reduction Agency, UNITED STATES

## Abstract

**Background:**

Sex and age strongly influence the pathophysiology of human lungs, but scarce information is available about their effects on pulmonary gene expression.

**Methods:**

We followed a discovery-validation strategy to identify sex- and age-related transcriptional differences in lung.

**Results:**

We identified transcriptional profiles significantly associated with sex (215 genes; FDR < 0.05) and age at surgery (217 genes) in non-involved lung tissue resected from 284 lung adenocarcinoma patients. When these profiles were tested in three independent series of non-tumor lung tissue from an additional 1,111 patients, we validated the association with sex and age for 25 and 22 genes, respectively. Among the 17 sex-biased genes mapping on chromosome X, 16 have been reported to escape X-chromosome inactivation in other tissues or cells, suggesting that this mechanism influences lung transcription too. Our 22 age-related genes partially overlap with genes modulated by age in other tissues, suggesting that the aging process has similar consequences on gene expression in different organs. Finally, seven genes whose expression was modulated by sex in non-tumor lung tissue, but no age-related gene, were also validated using publicly available data from 990 lung adenocarcinoma samples, suggesting that the physiological regulatory mechanisms are only partially active in neoplastic tissue.

**Conclusions:**

Gene expression in non-tumor lung tissue is modulated by both sex and age. These findings represent a validated starting point for research on the molecular mechanisms underlying the observed differences in the course of lung diseases among men and women of different ages.

## Introduction

In humans, males and females share a common genome, except for a relatively small number of genes on the Y chromosome; however, the two sexes are noticeably different in morphology, physiology, and behavior. Whole genome analysis of gene expression in different tissues has shown widespread sex differences in the transcriptional profiles of genes [1–3]. This phenomenon, called sex-biased gene expression, has been attributed to the existence of pleiotropic effects of sex on the regulation of gene expression [1–3]. A study in Drosophila has indeed shown that female-biased genes on chromosome X have pleiotropic effects [4].

In addition to sex, other biological factors that strongly influence gene expression are age and the process of aging. Indeed, in studies that used genome-wide analyses, age-related changes in transcriptome profiles have been observed in several human tissues, including kidney, muscle, brain, skin, blood and adipose tissue [5–8]. However, there is still open debate on whether the aging process causes similar transcriptional changes in all tissues, and, therefore, if it is possible to identify a common aging signature, or if it predominantly induces tissue-specific molecular changes. Indeed, one study found that only a few genes are commonly affected by age in skin, adipose and brain [6], whereas another found six common age-modulated pathways in a comparison of muscle, kidney and brain [7]. Finally, a meta-analysis of multiple tissues from humans, rats and mice identified common signatures of aging involving, for example, immune/inflammatory responses, cell growth, energy metabolism, and extracellular matrix components [8].

In lung, the effects of age and sex on gene expression are just beginning to be investigated. One study of post-mortem lung tissue from multiorgan donors found 40 genes that were differentially expressed between 7 young and 6 old persons and obtained preliminary results about sex-related differences in gene expression [9]. Substantially more information is available about the effects of age and sex on lung function and cellular physiology. In general, pulmonary function declines with age, even in the absence of respiratory disease. The elderly population has decreased lung volumes, less efficient functionality of respiratory muscles and reduced forced expiratory volumes, accompanied by modifications in innate and adaptive immunity (reviewed in [10]). Lung anatomy and physiology as well as the etiology of some respiratory diseases (e.g. asthma, allergic rhinitis, pulmonary hypertension, cystic fibrosis) have been reported to show sex differences, from prenatal lung development to adulthood; some studies suggested a possible role of sex hormones to explain these differences, but sociocultural and genetic differences between the sexes are also thought to be involved (reviewed in [11]).

In lung cancer, there are conflicting data on the effects of age and sex. Two large cohort studies reported better survival in women than men [12,13]. Age at diagnosis did not associate with survival in surgically treated patients with stage I non-small cell lung cancer [14]. We previously observed that neither age at diagnosis nor sex was significantly associated with survival in patients with lung adenocarcinoma [15]. In light of this uncertainty, and considering the relevance of age and sex on lung function in health and disease, greater knowledge about the genes modulated by these biological parameters in lung tissue is needed. Such information is crucial for understanding the molecular mechanisms underlying the observed physiological differences among individuals in the absence of disease, and may also help uncover new targets for the treatment of lung pathologies.

In the present work, we studied the role of sex and age on gene expression in lung tissue, by doing a statistical reanalysis of existing microarray data. We first identified genes affected by sex and age in samples of non-involved lung tissue resected from men and women with lung adenocarcinoma over a broad range of ages (discovery series, [15]). We then determined which of these genes were also differentially expressed in three other series of non-tumor lung tissue (validation series, [16]). Finally, we examined public datasets of gene expression in lung tumors to determine if the sex- and age-biased transcriptomic profiles observed in non-tumor lung tissue are also detectable in lung cancer tissue.

## Materials and Methods

### Ethics statement

The present study used existing microarray data from four independent clinical series, already described [15,16]. Those papers detailed the approval of the study protocols by the institutional ethics committees (Fondazione IRCCS Istituto Nazionale dei Tumori, Milan, Italy, Istituto Clinico Humanitas, Rozzano, Italy, Ospedale San Giuseppe, Milan, Italy, Fondazione IRCCS Cà Granda Ospedale Maggiore Policlinico, Milan, Italy, Institut Universitaire de cardiologie et de pneumologie de Québec, Québec, Canada, University of British Columbia, Vancouver, BC, Canada, University of Groningen, Groningen, The Netherlands) and the collection of surgical samples and clinical data from patients who had provided broad informed consent allowing the materials to be used for research purposes; even though follow-up studies were not explicitly authorized, neither were they explicitly refused, and it was expected by all parties involved that the samples and data would support multiple studies. At the recruitment, the authors got the data that have been, then, used in this study. The current analysis did not require additional ethical approval, because it is a continuation of the originally approved studies on data that were full available prior to their use in this study.

### Discovery series

The discovery series consisted of 284 samples of non-involved (apparently normal) lung parenchyma (sampled as far as possible from tumor tissue), and the associated clinical data, from patients who had had lobectomy for lung adenocarcinoma at the Fondazione IRCCS Istituto Nazionale dei Tumori and at other hospitals in the area of Milan, Italy. Details about sample collection, RNA extraction and gene expression profiling on Illumina HumanHT-12 v4 Expression BeadChips have already been reported [15]. Because that study also had a discovery-validation design, the microarray analysis had been done on consecutive sets of 206 and 78 samples (although 2 samples from the first set had been excluded from analysis due to the lack of follow-up data).

The analysis of raw microarray data was done at Fondazione IRCCS Istituto Nazionale dei Tumori in Milan essentially as previously described [15]. Briefly, the two datasets of 206 and 78 samples were processed independently using log_2_ transformation and robust spline normalization, implemented in the *lumi* package [17] of the open source software Bioconductor [18]. Then, the two sets were combined using the ComBat adjustment method [19] implemented in the *sva* R package [20]. Probes that were not annotated and those with a detection *P* value < 0.01 in fewer than 10% of samples were filtered out. Finally, when multiple probes mapped to the same transcript, we included only the one with the highest detection rate, defined as the percentage of samples in which the probe had detection *P* values < 0.01. Gene expression data were deposited in the Gene Expression Omnibus database (GEO, http://www.ncbi.nlm.nih.gov/geo/) with accession number GSE71181.

### Validation series

This study also used three validation series, consisting of the discovery and replication sets of another previous work [16]. Here, those sets are called the Laval (n = 409), University of British-Columbia (UBC; n = 339) and Groningen (n = 363) series according to the names of the sites were the patients had lung resection surgery, namely Laval University (Quebec, Canada), University of British-Columbia (Vancouver, Canada), and University of Groningen (Groningen, The Netherlands), respectively. In all cases, non-tumor lung tissues were sampled.

The previous publications [16,21] detailed the sample collection, RNA extraction and gene expression profiling on custom Affymetrix arrays for all three series. Briefly, expression values were extracted from microarray data using the robust multichip average (RMA) method [22]. When two or more probe sets mapped to the same transcript, the one with the greatest number of present calls was selected. The resulting microarray data were deposited in the GEO database with accession number GSE23546.

### Datasets on gene expression in lung cancer

To further validate our findings we downloaded, from the GEO database, five public datasets (GSE68896, GSE30219, GSE31210, GSE37745, and GSE41271). GSE68896 contains normalized gene expression data from fetal lung tissue, whereas GSE30219, GSE31210, GSE37745, and GSE41271 contain normalized gene expression data from patients with lung cancer for whom data about age at surgery and sex were available (**S1 Table**). From each lung cancer dataset, we discarded the records about normal lung tissue samples.

### Statistical analysis

Associations of gene expression data with sex and age at surgery (used as dichotomous and continuous variables, respectively) were tested using linear regression modeling. Smoking habit was used as a covariate in the validation series but not in the discovery series, as all samples except one were from smokers, nor in the public lung cancer tissue datasets which lacked information about smoking status.

In the validation phase, the two sets of sex- and age-associated genes were tested independently in each validation series. In both discovery and validation phase, all *P*-values were corrected for multiple testing using the Benjamini-Hochberg false discovery rate (FDR) method [23]; the threshold for statistical significance was set at FDR < 0.05.

To identify sex- and age-biased known biological pathways, we ran gene set enrichment analysis (GSEA) using GSEA v2.0.13 software [24]. Results were visualized using Cytoscape v2.8.3 software [25] and the Enrichment Map plugin [26].

## Results

**Fig 1:**
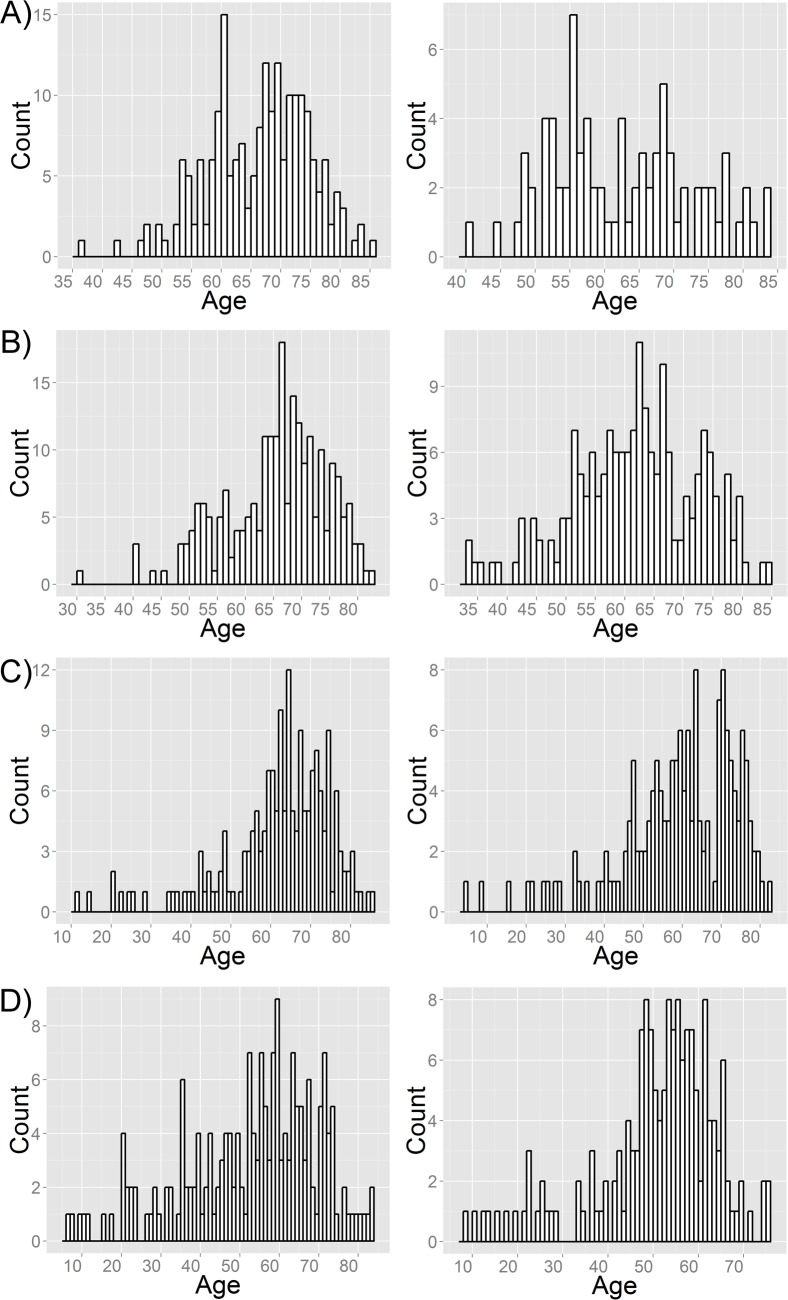
Age distributions of discovery and validation series. Histogram of age at surgery of male (left panels) and female (right panels) lung cancer patients of the discovery (A) and validation series (B: Laval University; C: University of British Columbia; D: University of Groningen).

To study the effects of sex and age on gene expression in lung tissue, we analyzed a discovery series of 284 surgical samples from Italy [15] and three validation series from Canada and the Netherlands [16] (**Table 1**). The discovery series had a predominance of men (71.1%) and all but one were ever-smokers. The validation series had a more balanced sex distribution, with men representing 53%–56%, and included patients who were never-smokers as well as current and former smokers. In the discovery series, age of both male (**Fig 1A**, left) and female (**Fig 1A**, right) patients showed a wide distribution, ranging from 36 to 85 years in males (median = 67 years) and from 40 to 83 years in females (median = 62 years). Age also showed a wide distribution in the three validation sets. For Laval, age ranged from 30 to 82 years in males (**Fig 1B**, left, median = 66 years) and from 33 to 84 years in females (**Fig 1B**, right, median = 62 years). For UBC, age ranged from 11 to 85 years in males (**Fig 1C**, left, median = 64 years) and from 4 to 82 years in females (**Fig 1C**, right, median = 61 years). For Groningen, age ranged from 6 to 83 years in males (**Fig 1D**, left, median = 56 years) and from 8 to 75 years in females (**Fig 1D**, right, median = 53 years).

**Table 1:** Clinical characteristics of patients from whom a sample of resected non-tumor lung tissue was included in the discovery and validation series.

	Discovery Series (n = 284)	Validation series		
		Laval (n = 409)	UBC (n = 339)	Groningen (n = 363)
Gender				
Men	202 (71.1)	229 (56.0)	182 (53.7)	193 (53.2)
Women	82 (28.9)	180 (44.0)	157 (46.3)	170 (46.8)
Age at surgery, years, mean (SD)	64.7 (9.2)	63.3 (9.9)	60.2 (14.3)	51.5 (15.5)
Smoking status				
Current smoker	283 (99.7)^a^	90 (22.0)	98 (28.9)	57 (15.7)
Former smoker	-^a^	283 (69.2)	163 (48.1)	185 (51.0)
Never-smoker	1 (0.3)	36 (8.8)	26 (7.7)	100 (27.5)
Unknown	0 (0)	0 (0)	52 (15.3)	21 (5.8)
Reason for surgery				
Lung cancer	284 (100)	408 (99.8)	274 (80.8)	123 (33.9)
Other	0 (0)	1 (0.2) ^b^	65 (19.2) ^b^	240 (66.1) ^b^
Lung cancer histotype				
Adenocarcinoma	284 (100)	235 (57.6)	88 (32.1)	37 (30.1)
Squamous cell carcinoma	0 (0)	104 (25.5)	86 (31.4)	54 (43.9)
Carcinoid	0 (0)	17 (4.2)	15 (5.5)	2 (1.6)
Small cell lung carcinoma	0 (0)	7 (1.7)	23 (8.4)	2 (1.6)
Other	0 (0)	45 (11.0)	62 (22.6)	28 (22.8)

Values are n (%) unless indicated otherwise.

^a^ In the discovery series, individuals are categorized as either never-smokers or ever-smokers, without distinguishing the latter into current smokers or ex-smokers.

^b^ Patients underwent therapeutic lung resection for lung transplantation or for small peripheral lung lesions.

All patients in the discovery series had been treated for lung adenocarcinoma, while the validation series included patients with a variety of different lung cancer types and even patients who had other lung diseases. In all cancer cases, the surgical samples were apparently normal, having been taken from the non-diseased margins of the resected tissue. Considering the reasons for which the patients had lung resection, their smoking habits and their ages at surgery, the Laval validation series was the most similar to the discovery series.

### Discovery of transcripts in lung tissue that associate with sex and age

Expression data were available regarding 11,089 genes for the discovery series [15]. Of these, 215 genes (2%) were differentially expressed between men and women (**S2 Table**). In particular, 122 genes were up-regulated and 93 genes were down-regulated in men compared to women (FDR < 0.05). All genes had a fold change between -2 and 2 except for two: *RPS4Y1*, mapping on chromosome Y, was 48-fold up-regulated in men (compared to the background probe intensity measured in females, who do not carry the gene), while *XIST*, mapping on chromosome X, was 12-fold down-regulated. Altogether, 30 sex-associated genes mapped to the non-pseudoautosomal region of chromosome X, one mapped to the non-pseudoautosomal region of chromosome Y, four mapped to the pseudoautosomal regions, and the remaining 180 genes mapped to autosomes. To determine if these genes distinguishing the normal lung tissue of male and female individuals were associated with particular biological pathways or functions, we performed gene set enrichment analysis (GSEA) [24] using gene sets from the Gene Ontology collection (C5.all.v4.0, retrieved from http://www.broadinstitute.org/gsea/msigdb/index.jsp). Despite the large number of genes affected by sex, no gene set was found to be enriched in male vs. female individuals.

**Fig 2:**
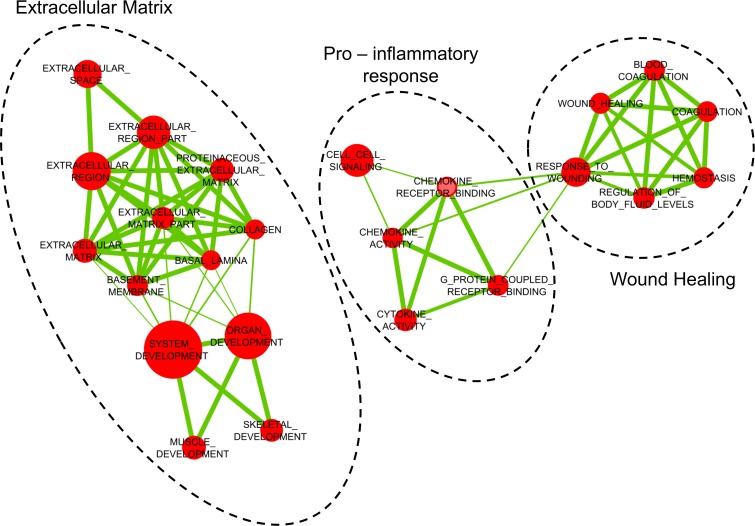
Two major functional themes displayed an altered expression in older patients. Network-based visualization of gene sets enriched in lung tissue in association with age, in 284 patients who underwent lobectomy for lung adenocarcinoma; the analyzed lung tissue was not involved by cancer. Gene set enrichment analysis (GSEA) was carried out using GSEA v2.0.13 software [24]. Briefly, we first ranked genes of the discovery dataset according to the t-statistic of age and sex. Then, gene sets derived from the Gene Ontology database (C5.all.v4.0) were retrieved from the Molecular Signature Database (MSigDB, http://www.broadinstitute.org/gsea/msigdb/index.jsp) and tested for enrichment using a Kolmogorov-Smirnov statistic. Only the 653 gene sets having between 15 and 500 genes were tested for enrichment. An FDR < 0.05 identified significantly enriched gene sets. GSEA results were visualized using Cytoscape v2.8.3 software [25] and the Enrichment Map plugin [26], with an overlap coefficient cut-off of 0.5. Circles (nodes) represent C5 Gene Ontology gene sets connected by lines (edges) whose thickness is proportional to the number of genes shared between the connected nodes. Circle sizes are proportional to the number of genes annotated in each gene set. Two independent clusters of functionally related gene sets were detected, one (on the left) involving extracellular matrix and the other one (on the right) involving pro-inflammatory response and wound healing.

We then examined the effect of age at surgery on gene expression and found 217 genes that were significantly associated with patients’ age (FDR < 0.05; **S3 Table**). In particular, the expression of 124 genes was positively associated with age, while 93 genes showed a decreased expression in older patients. GSEA revealed that two major functional themes displayed an altered expression in older patients (**Fig 2**). The first included gene sets related to extracellular matrix components and functions, with genes encoding, for example, collagens, laminins and metallo-proteinases. The second comprised gene sets related to pro-inflammatory responses and wound healing.

In order to evaluate a possible effect of chance in finding a number of sex- or age-associated genes similar to those observed in this study, we performed a permutation analysis on the discovery series by randomly assigning sex and age labels for 1,000 times; at each permutation cycle, we calculated: i) the total number of genes with FDR < 0.05, to determine the expected number of significant genes occurring by chance; ii) the number of times each of the 215 sex-related and 217 age-related genes had an FDR < 0.05. We found that in 969 out of 1,000 permutations no genes were significantly associated with sex, with an expected number of genes by chance of 0.721. Results were similar for age, with 972 of 1,000 times with no significant genes and with an expected number of genes by chance of 1.817. This analysis clearly indicates that the limited number of genes that came out of the analysis was not casually generated.

### Validation of the sex-biased transcriptional profile

**Fig 3:**
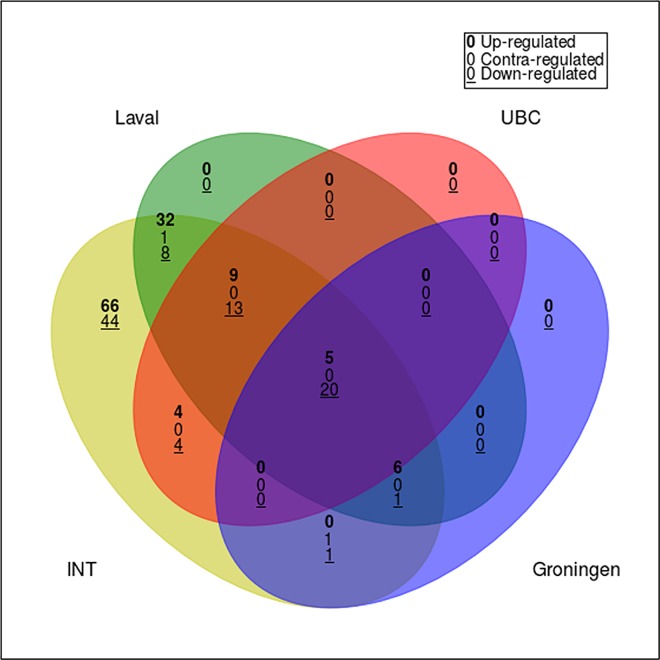
Intersection of the lists of genes significantly associated with sex in each of the four datasets. Four-way Venn diagram analysis of sex-related transcriptional alterations in non-tumor lung tissue. Each of the circles depicts the number of different transcripts based on a sex comparison for each of the labeled data series (yellow, INT, Italian discovery series; green, Laval validation series; blue, Groningen validation series; red, UBC validation series) among the 215 transcripts identified as statistically significant in the Italian discovery series. Shared transcripts are represented in the areas of intersection between two or more circles. Genes whose expression levels were positively and negatively associated with sex in the different series are called “contra-regulated”; these genes were not included among the number of validated genes.

We then attempted to validate the results from the discovery series in three independent validation series (Laval, n = 409; UBC, n = 339; and Groningen, n = 363) [16]. Of the 215 genes associated with sex in the discovery phase, six were not represented on the platform used to profile the validation datasets. For the remaining 209 genes, the possibility of false-positive results due to the high number of statistical tests carried out was taken into account by correcting the obtained *P*-values for multiple testing with the Benjamini–Hochberg procedure and setting a significance threshold of FDR < 0.05. With this analysis, 94 genes in the Laval series showed statistically significant and concordant associations with sex and were therefore validated. In addition, 55 genes in the UBC series and 33 in the Groningen series were also validated. Intersection of the lists of genes significantly associated with sex in each of the four datasets (discovery, Laval, UBC and Groningen) highlighted a common set of 25 genes that were validated in all series (**Fig 3**).

This common set of sex-biased genes in non-tumor lung tissue included five genes that were up-regulated and 20 that were down-regulated in men compared to women. Overall, five of the genes mapped to four different autosomes, 17 mapped to the X chromosome and one to the Y chromosome (pseudo-autosomal regions excluded), and two mapped to the pseudoautosomal regions (**Table 2**). The two genes that showed an absolute fold change greater than 2 between males and females in the discovery series (*RPS4Y1* and *XIST*) were included in this set.

**Table 2:** Genes whose expression in lung tissue associated with sex in the discovery series and in all three validation series.

Gene symbol	Gene name	Gene Entrez ID	Chr.	Fold change ^1^	FDR ^1^
*AOX1*	Aldehyde oxidase 1	316	2	1.397	2.81E-04
*ARSD*	Arylsulfatase D	414	X	-1.543	5.78E-50
*ATP6V1B1*	ATPase, H+ transporting, lysosomal 56/58kDa, V1 subunit B1	525	2	-1.352	3.30E-04
*CD99*	CD99 molecule	4267	X;Y	1.342	5.85E-22
*CILP*	Cartilage intermediate layer protein, nucleotide pyrophosphohydrolase	8483	15	1.397	1.24E-02
*DDX3X*	DEAD (Asp-Glu-Ala-Asp) box helicase 3, X-linked	1654	X	-1.215	2.29E-04
*EIF1AX*	Eukaryotic translation initiation factor 1A, X-linked	1964	X	-1.336	1.34E-16
*EIF2S3*	Eukaryotic translation initiation factor 2, subunit 3 gamma, 52kDa	1968	X	-1.231	1.39E-16
*GEMIN8*	Gem (nuclear organelle) associated protein 8	54960	X	-1.134	1.20E-04
*HDHD1*	Haloacid dehalogenase-like hydrolase domain containing 1	8226	X	-1.586	1.75E-50
*KAL1*	Kallmann syndrome 1 sequence	3730	X	-1.982	1.75E-30
*KDM6A*	Lysine (K)-specific demethylase 6A	7403	X	-1.514	4.96E-39
*OFD1*	Oral-facial-digital syndrome 1	8481	X	-1.179	5.06E-04
*OLFML2A*	Olfactomedin-like 2A	169611	9	-1.287	6.86E-03
*PHGDH*	Phosphoglycerate dehydrogenase	26227	1	-1.170	3.64E-02
*PRKX*	Protein kinase, X-linked	5613	X	-1.131	2.38E-03
*RPS4X*	Ribosomal protein S4, X-linked	6191	X	-1.369	1.83E-09
*RPS4Y1*	Ribosomal protein S4, Y-linked 1	6192	Y	48.948	1.76E-249
*TRAPPC2*	Trafficking protein particle complex 2	6399	X	-1.171	1.72E-07
*TXLNG*	Taxilin gamma	55787	X	-1.184	7.33E-07
*USP9X*	Ubiquitin specific peptidase 9, X-linked	8239	X	-1.084	2.15E-02
*XIST*	X inactive specific transcript (non-protein coding)	7503	X	-12.561	1.14E-171
*ZBED1*	Zinc finger, BED-type containing 1	9189	X;Y	1.108	3.91E-05
*ZFX*	Zinc finger protein, X-linked	7543	X	-1.382	3.14E-21
*ZRSR2*	Zinc finger (CCCH type), RNA-binding motif and serine/arginine rich 2	8233	X	-1.362	2.54E-16

^1^ Values are those observed in the discovery series; males versus females, values > 1 indicate higher levels in males.

To assess whether transcriptional differences in the lung of males and females arise at early stages of development, we analyzed public gene expression data of fetal lung tissues (GSE68896, downloaded from the Gene Expression Omnibus database at http://www.ncbi.nlm.nih.gov/geo/). Interestingly, we found that 18 of the 25 validated sex-related genes identified in adult lung tissue (**Table 2**) were also differentially expressed between male and female fetal lung tissues (**S4 Table**).

We also attempted to validate the results from the discovery series in four publicly available datasets of gene expression in lung cancer samples (GSE30219, GSE31210, GSE37745, and GSE41271). As these four datasets were profiled with different platforms, data were available for 169 of the 215 genes associated with sex in the discovery series. Among these 169 genes, 52 showed a statistically significant (FDR < 0.05) and concordant association with sex in at least one cancer dataset (not shown). The discovery series and the four tumor series shared a common set of seven sex-biased genes, namely *ARSD*, *CD99*, *GEMIN8*, *OFD1*, *RPS4X*, *RPS4Y1*, and *XIST*. Interestingly, all of these genes also belonged to the common set of genes validated in all three series of non-tumor lung tissue.

### Validation of the age-biased transcriptional profile

**Fig 4:**
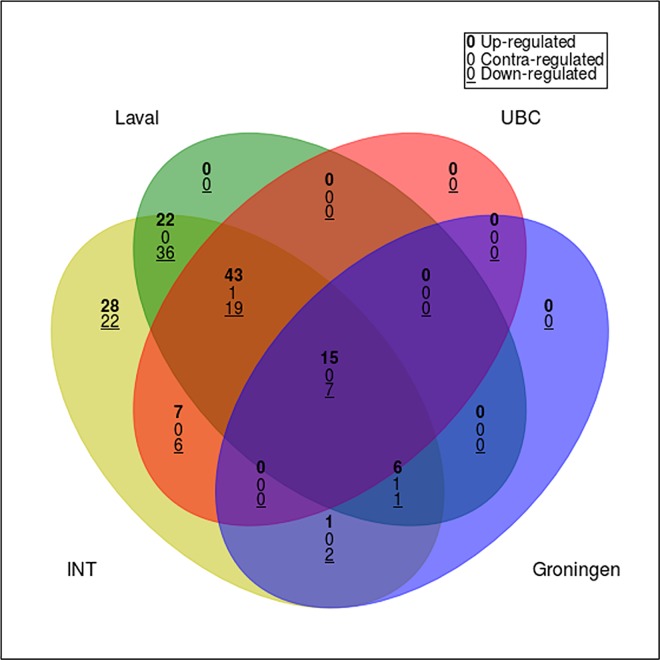
Intersection of the lists of genes significantly associated with age in each of the four datasets. Four-way Venn diagram analysis of age-related transcriptional alterations in non-tumor lung tissue. As in Fig 3, each of the circles depicts the number of different transcripts based on age comparison for each of the labeled data series (yellow, INT, Italian discovery series; green, Laval validation series; blue, Groningen validation series; red, UBC validation series) among the 217 transcripts identified as statistically significant in the Italian discovery series. Shared transcripts are represented in the areas of intersection between two or more circles. Genes whose expression levels were positively and negatively associated with age in the different series are called “contra-regulated”; these genes were not included among the number of validated genes.

Among the 217 genes associated with age at surgery in the discovery series, 13 had not been investigated in the three validation series (Laval, UBC, Groningen). After correcting for multiple testing, the association with age (with the same direction of effect) was confirmed for 149 genes in the Laval series, 97 genes in the UBC series, and 32 genes in the Groningen series (FDR < 0.05). The intersection of these sets identified 22 common genes showing a concordant direction of effect in all four series (**Fig 4**). These included 15 up-regulated and 7 down-regulated genes in older individuals, which were found on 14 different autosomes (**Table 3**).

**Table 3:** Genes whose expression in tissue associated with patients’ age at surgery in the discovery series and in all three validation series.

Symbol	Gene name	Gene Entrez ID	Chr.	Estimate ^1^	FDR ^1^
*AGR2*	Anterior gradient 2	10551	7	-0.01213	2.95E-02
*CRYAB*	Crystallin, alpha B	1410	11	0.013824	1.60E-03
*CXCL17*	Chemokine (C-X-C motif) ligand 17	284340	19	-0.00911	4.85E-02
*CXCL9*	Chemokine (C-X-C motif) ligand 9	4283	4	0.028698	3.35E-02
*DIRAS3*	DIRAS family, GTP-binding RAS-like 3	9077	1	0.008936	3.00E-03
*FANCE*	Fanconi anemia, complementation group E	2178	6	-0.01075	1.10E-03
*FMO3*	Flavin containing monooxygenase 3	2328	1	0.0131	1.44E-02
*GSTA4*	Glutathione S-transferase A4	2941	6	-0.01173	4.80E-03
*ITGB5*	Integrin, beta 5	3693	3	0.007076	4.19E-02
*ITGBL1*	Integrin, beta-like 1 (with EGF-like repeat domains)	9358	13	0.018297	1.21E-05
*LCN2*	Lipocalin 2 (oncogene 24p3)	3934	9	-0.02576	3.78E-02
*MGP*	Matrix Gla protein	4256	12	0.00841	4.97E-02
*PGM5*	Phosphoglucomutase 5	5239	9	0.009683	4.80E-02
*PLCD3*	Phospholipase C, delta 3	113026	17	0.005974	1.42E-02
*PTCHD4*	Patched domain containing 4	442213	6	0.003035	4.85E-02
*RCAN2*	Regulator of calcineurin 2	10231	6	0.015413	1.56E-04
*RERGL*	RERG/RAS-like	79785	12	0.022899	6.70E-03
*SCARF2*	Scavenger receptor class F, member 2	91179	22	0.009692	1.96E-02
*TC2N*	Tandem C2 domains, nuclear	123036	14	-0.00923	3.78E-02
*WISP2*	WNT1 inducible signaling pathway protein 2	8839	20	0.026326	9.02E-05
*ZMAT3*	Zinc finger, matrin type 3	64393	3	0.016636	1.84E-04
*ZNF518B*	Zinc finger protein 518B	85460	4	-0.00626	4.07E-02

^1^ Values are those observed in the discovery series.

The same analysis was repeated for the four public datasets of lung cancer specimens (GSE30219, GSE31210, GSE37745, and GSE41271). Of the 217 age-associated genes found in the discovery series, 178 had also been investigated in the four tumor series. Statistical analysis showed that only one gene (*IRX3*) maintained a significant (FDR < 0.05) and concordant association in at least one of the four cancer datasets (not shown), and none maintained the association in all four datasets. Therefore, it was not possible to identify a common set of genes regulated by age in lung tumor tissue.

## Discussion

In our discovery series of 284 lung adenocarcinoma patients, we identified 215 and 217 genes whose expression levels in non-involved lung tissue were significantly associated with sex and age, respectively. GSEA analysis revealed that no specific molecular functions were over-represented among the genes altered in males vs. females, whereas genes whose expression was altered in older patients belonged to two major functional categories, i.e. extracellular matrix function and pro-inflammatory response/wound healing. We validated 25 out of 215 sex-related genes and 22 out of 217 age-related genes in three independent series of non-tumor lung tissue (from a total of 1,111 individuals). Additionally, we validated a common set of seven sex-biased genes in four publicly available datasets of transcriptional profiles of lung tumor tissue (990 total individuals); no common age-associated genes were identified in all four lung cancer datasets, and only one gene was validated in at least one of the four tumor series.

Regarding the effect of sex on lung tissue transcription, we observed that 17 of the 25 sex-biased genes map to the non-pseudoautosomal region of chromosome X and displayed higher expression in females. These genes have probably escaped X-chromosome inactivation, i.e. the mechanism by which mammals balance gene expression between the sexes through the silencing of one X chromosome in somatic cells of females [27]. Indeed, all these genes have been observed to escape X-chromosome inactivation in an *in vitro* assay using rodent/human somatic cell hybrids [28], with the exception of *ZRSR2* which was not tested in that study. X-chromosome inactivation is regulated by several factors, including the *XIST* gene which we found to be highly up-regulated (12 fold) in females. Furthermore, four genes identified here (*HDHD1*, *RPS4X*, *KDM6A*, and *ZFX*) were found to have higher expression in females in various human tissues [29], although in that study only *KDM6A* (alias *UTX*) was differentially expressed in lung tissue. We also observed the sex-biased expression of genes never reported to escape from X-inactivation in human tissue and also of genes mapping on autosomes, suggesting the existence of other mechanisms of sex-dependent transcriptional control in lung tissue. As we did not find any functional category enriched among male- or female-specific genes, we cannot hypothesize about the mechanisms involved. Of note, in our analysis we did not consider the possible confounding effects of hormonal status on gene expression. Due to the small number (n = 8 women younger than 50 years of age) of non- or pre-menopausal females in our series, we doubt that menopausal status had any relevant impact on our results. Indeed, any subgroup analysis based on age in females would be severely biased by a very small sample size in one subgroup. Finally, we also noticed that >70% (i.e., 18/25) of the sex-related genes that passed validation showed a differential expression in males and females already in fetal lung, indicating that sexual differences in lung transcriptome are, at least in part, established at the developmental level.

Regarding age-dependent modulation of transcription, our study builds on preliminary evidence [9] of age-related changes in gene expression in non-tumor lung tissue and defines a lung-specific aging signature of 22 genes. Only the *HEPH* gene of our 22 genes was also present in the preliminary age-related signature of 40 genes reported in [9]. Five genes in our signature have previously been associated with aging in skin and/or adipose tissue [6]. In particular, two of them (*DIRAS3*, and *WISP2*) were found to be up-regulated in aged skin; two genes (*CXCL9* and *RCAN2*) were up-regulated with age in adipose tissue; and *FMO3* was reported up-regulated in both aged skin and adipose tissue. Their involvement in aging lung, reported here, suggests that these genes may have broad roles in the aging process.

None of the 22 age-related genes identified in this study belongs to the transcriptional signature of aging defined in a meta-analysis of multiple tissues from humans, rat and mice [8]. That study identified age-dependent modifications in the expression of genes involved in several biological processes, including pro-inflammatory response genes, and extracellular matrix function. We, too, found age-related changes in the expression of genes involved in pro-inflammatory responses and genes encoding extracellular matrix components, in our discovery series. Although there were no common genes between our study and the meta-analysis described in [8], pathways related to the pro-inflammatory responses and to the extracellular matrix are important in lung function and disease. The known age-related increase in pulmonary inflammation, as observed for example in lungs of patients with chronic obstructive pulmonary disease (COPD) [30], may reflect the lungs’ exposure, over the course of a lifetime, to environmental pollutants and microorganisms. Also, age-related changes in extracellular matrix alter the mechanisms of lung repair and lead to abnormal wound healing and fibrosis (reviewed in [31]). Such alterations are often observed, respectively, in COPD and in idiopathic pulmonary fibrosis, both associated with aging [32,33]. Therefore, the age-related genes identified in our study are worthy of further investigation to understand their possible involvement in these pulmonary diseases.

Interestingly, seven genes belonging to the sex-associated profile from non-involved lung tissue were also validated in four lung cancer series. This finding suggests that some sex-related transcriptome changes are maintained in neoplastic tissue which, therefore, is controlled to a certain degree by the same sex-linked factors active in normal lung tissue. In the same four lung cancer series, however, we did not validate any genes from the age-related expression profile and only one gene showed a significant association with age in at least one of the four datasets. This result suggests that gene expression in lung tumor tissue is not affected by factors associated with aging in non-tumor tissue.

One limitation of our study is that we do not have more details about patients’ smoking habits (e.g. years of smoking, packs per year) in the discovery series; thus we could not take into account this aspect in our analyses. Also, the study was carried out in whole lung tissue; therefore, the cell-specificity of expression of the validated genes should be further investigated by immunohistochemical studies.

Two other limitations of our study, which might explain the low number of validated sex- and age-related genes, are the use of different platforms for gene expression analysis in the discovery versus validation series, and the high degree of heterogeneity, in terms of patients’ ages, sex distribution, smoking habits, and types of lung pathology, of the validation series compared with the discovery series. Indeed, the number of validated genes is quite low (around 10% of those found in the discovery series for both sex and age), but the validation was carried out on three independent datasets. Because this approach is stringent, it results in a lower number of validated genes than had we used only a single validation dataset, as seen in the majority of published reports. The relatively few genes validated in our study represent the core of genes that are always significantly associated with sex or age in all of the datasets analyzed; we believe that these are true positives. If we are less stringent and consider the genes found significant in at least one dataset, the numbers of validated genes increase from 25 to 102 for sex and from 22 to 165 for age.

It is interesting to notice that more genes were validated for sex and age in the Laval series, which is clinically most similar to the Italian discovery series, than in the other two validation series. This finding suggests that the transcriptional profiles identified in the Italian discovery series contain a high percentage of real positive associations. This is also supported by permutation analysis results indicating a very low possibility that the obtained numbers of sex- or age-related genes were due to chance.

The mechanisms underlying the observed differential sex or age expression of genes in lung may be due to differences in DNA methylation. Indeed, sex differences in DNA methylation, leading to differential gene expression at various loci, have been reported [34]. Moreover, age-related changes in DNA methylation have also been observed [35].

Overall, we found that, in non-tumor lung tissue, several genes undergo a modulation of their expression, which depends on either sex or age of the examined individual. Our findings provide a reliable starting point for a deeper investigation at the molecular level of the role of sex and age in the pathophysiology of lung tissue.

## Supporting Information

S1 Table Clinical characteristics of patients with lung cancer whose resected tumor tissue was analyzed for gene expression, according to the GEO dataset in which the data were deposited (DOCX)

S2 Table List of 215 genes whose expression in lung tissue associated with gender in the discovery series, in order of false discovery rate (FDR) values. (DOCX)

S3 Table List of 217 genes whose expression in lung tissue associated with age at surgery in the discovery series, presented in order of false discovery rate (FDR) values. (DOCX)

S4 Table Sex-related differentially expressed genes in both adult and fetal lung tissue. Of the 25 validated sex-related genes identified in adult lung tissue, 18 were also found to be differentially expressed between males and females in fetal lung tissue (GSE68896)
(DOCX)
