# Supplementary material for: Human Lung Tissue Transcriptome: Influence of Sex and Age
Source: PLoS One. 2016 Nov 30;11(11):e0167460. doi: 10.1371/journal.pone.0167460 (PMC5130276; doi:10.1371/journal.pone.0167460)
Supplement: S1 Table — (DOCX) [file pone.0167460.s001.docx]

**S1 Table.** Clinical characteristics of patients with lung cancer whose resected tumor tissue was analyzed for gene expression, according to the GEO dataset in which the data were deposited

|  | **GSE30219**  **(n=293)** | **GSE31210**  **(n=226)** | **GSE37745**  **(n=196)** | **GSE41271**  **(n=275)** |
| --- | --- | --- | --- | --- |
| Gender |  |  |  |  |
| Men (%) | 250 (81) | 105 (46) | 107 (55) | 148 (54) |
| Women (%) | 43 (19) | 121 (54) | 89 (45) | 127 (46) |
| Age at surgery, years, median (range) | 62.5 (15-84) | 61 (30-76) | 65 (39-84) | 65 (30-86) |
| Smoking status |  |  |  |  |
| Current smoker | - | 111 (49)* | - | 244 (90)* |
| Former smoker | - | -* | - |  |
| Never-smoker | - | 115 (51) | - | 28 (10) |
| Unknown | 293 | 0 | 196 | 0 |
| Lung cancer histotype |  |  |  |  |
| Adenocarcinoma | 85 | 226 | 106 | 183 |
| Squamous cell carcinoma | 61 | 0 | 66 | 80 |
| Carcinoid | 24 | 0 | 0 | 0 |
| Small cell lung carcinoma | 21 | 0 | 0 | 0 |
| Other | 102 | 0 | 24 | 12 |

Values are n (%) unless otherwise indicated.

* For the GSE31210 and GSE41271 datasets, individuals are not distinguished between current and former smokers.
