# Supplementary material for: Human Lung Tissue Transcriptome: Influence of Sex and Age
Source: PLoS One. 2016 Nov 30;11(11):e0167460. doi: 10.1371/journal.pone.0167460 (PMC5130276; doi:10.1371/journal.pone.0167460)
Supplement: S2 Table — (DOCX) [file pone.0167460.s002.docx]

**S2 Table.** List of 215 genes whose expression in lung tissue associated with gender in the discovery series, in order of false discovery rate (FDR) values.

| Gene symbol | Chromosome | Fold change (males vs. females) | *P*-value | FDR |
| --- | --- | --- | --- | --- |
| *RPS4Y1* | Y | 48.948 | 1.58E-253 | 1.76E-249 |
| *XIST* | X | -12.561 | 2.05E-175 | 1.14E-171 |
| *HDHD1* | X | -1.585 | 4.75E-54 | 1.75E-50 |
| *ARSD* | X | -1.543 | 2.08E-53 | 5.78E-50 |
| *KDM6A* | X | -1.513 | 2.24E-42 | 4.96E-39 |
| *KAL1* | X | -1.982 | 9.46E-34 | 1.75E-30 |
| *CD99* | X;Y | 1.342 | 3.70E-25 | 5.85E-22 |
| *ZFX* | X | -1.382 | 2.27E-24 | 3.14E-21 |
| *EIF1AX* | X | -1.336 | 1.09E-19 | 1.34E-16 |
| *EIF2S3* | X | -1.231 | 1.25E-19 | 1.39E-16 |
| *ZRSR2* | X | -1.362 | 2.52E-19 | 2.54E-16 |
| *RPS4X* | X | -1.369 | 1.98E-12 | 1.83E-09 |
| *TRAPPC2* | X | -1.171 | 2.02E-10 | 1.72E-07 |
| *GPR64* | X | -1.188 | 2.98E-10 | 2.36E-07 |
| *TXLNG* | X | -1.184 | 9.91E-10 | 7.33E-07 |
| *ZBED1* | X;Y | 1.108 | 5.64E-08 | 3.91E-05 |
| *FUNDC1* | X | -1.108 | 1.61E-07 | 0.0001 |
| *GEMIN8* | X | -1.134 | 1.95E-07 | 0.0001 |
| *CHM* | X | -1.198 | 2.20E-07 | 0.0001 |
| *DDX3X* | X | -1.215 | 4.12E-07 | 0.0002 |
| *AOX1* | 2 | 1.397 | 5.32E-07 | 0.0003 |
| *ATP6V1B1* | 2 | -1.352 | 6.55E-07 | 0.0003 |
| *SFRP2* | 4 | 1.695 | 9.86E-07 | 0.0005 |
| *SULF2* | 20 | 1.243 | 1.03E-06 | 0.0005 |
| *OFD1* | X | -1.179 | 1.14E-06 | 0.0005 |
| *WISP2* | 20 | 1.380 | 2.09E-06 | 0.0009 |
| *HSD11B1* | 1 | 1.229 | 2.37E-06 | 0.0010 |
| *TSKU* | 11 | 1.250 | 2.48E-06 | 0.0010 |
| *CTPS2* | X | -1.135 | 5.64E-06 | 0.0021 |
| *AEN* | 15 | 1.203 | 5.96E-06 | 0.0021 |
| *LOC100240735* | 12 | 1.143 | 5.79E-06 | 0.0021 |
| *ALDH1A2* | 15 | 1.302 | 6.33E-06 | 0.0022 |
| *PRKX* | X | -1.131 | 7.10E-06 | 0.0024 |
| *THBD* | 20 | 1.298 | 9.13E-06 | 0.0030 |
| *ADA* | 20 | 1.186 | 1.03E-05 | 0.0033 |
| *HSPB3* | 5 | 1.244 | 1.14E-05 | 0.0035 |
| *CRBN* | 3 | -1.126 | 1.34E-05 | 0.0040 |
| *FAM65B* | 6 | 1.255 | 1.70E-05 | 0.0050 |
| *FAM105B* | 5 | 1.098 | 1.79E-05 | 0.0051 |
| *MAP1LC3C* | 1 | -1.243 | 1.99E-05 | 0.0054 |
| *VNN2* | 6 | 1.361 | 1.95E-05 | 0.0054 |
| *DIRAS3* | 1 | 1.127 | 2.21E-05 | 0.0057 |
| *WNT5A* | 3 | -1.225 | 2.21E-05 | 0.0057 |
| *NFATC1* | 18 | 1.333 | 2.29E-05 | 0.0058 |
| *APOD* | 3 | 1.330 | 2.56E-05 | 0.0063 |
| *KANK4* | 1 | -1.243 | 2.71E-05 | 0.0064 |
| *ASMTL* | X;Y | 1.107 | 2.88E-05 | 0.0064 |
| *COL6A5* | 3 | -1.278 | 2.79E-05 | 0.0064 |
| *DNAJC12* | 10 | 1.144 | 2.74E-05 | 0.0064 |
| *PDLIM3* | 4 | 1.268 | 2.84E-05 | 0.0064 |
| *CD1E* | 1 | -1.177 | 3.06E-05 | 0.0066 |
| *CDK5RAP3* | 17 | -1.183 | 3.09E-05 | 0.0066 |
| *OLFML2A* | 9 | -1.287 | 3.28E-05 | 0.0069 |
| *FCN1* | 9 | 1.308 | 3.50E-05 | 0.0072 |
| *UAP1* | 1 | 1.256 | 3.74E-05 | 0.0075 |
| *PMEPA1* | 20 | 1.159 | 4.01E-05 | 0.0079 |
| *ISG20* | 15 | 1.186 | 4.17E-05 | 0.0080 |
| *RORC* | 1 | -1.210 | 4.15E-05 | 0.0080 |
| *FAM150B* | 2 | 1.408 | 4.28E-05 | 0.0081 |
| *C1QTNF1* | 17 | 1.322 | 5.47E-05 | 0.0101 |
| *PRPH2* | 6 | 1.079 | 5.61E-05 | 0.0102 |
| *MANSC1* | 12 | 1.080 | 5.95E-05 | 0.0105 |
| *ZNF431* | 19 | -1.124 | 5.88E-05 | 0.0105 |
| *EMP1* | 12 | 1.304 | 6.16E-05 | 0.0107 |
| *LIFR* | 5 | -1.253 | 6.57E-05 | 0.0109 |
| *MTRF1L* | 6 | 1.098 | 6.51E-05 | 0.0109 |
| *PLCXD1* | X;Y | 1.189 | 6.55E-05 | 0.0109 |
| *SFRP4* | 7 | 1.426 | 6.96E-05 | 0.0113 |
| *CILP* | 15 | 1.397 | 7.91E-05 | 0.0124 |
| *PLA1A* | 3 | 1.275 | 7.95E-05 | 0.0124 |
| *RAI2* | X | 1.126 | 7.85E-05 | 0.0124 |
| *HBA2* | 16 | 1.282 | 8.30E-05 | 0.0125 |
| *IL17D* | 13 | 1.156 | 8.25E-05 | 0.0125 |
| *PRPS1* | X | 1.094 | 8.34E-05 | 0.0125 |
| *EBAG9* | 8 | -1.115 | 8.54E-05 | 0.0125 |
| *NLRP3* | 1 | 1.299 | 8.58E-05 | 0.0125 |
| *C22orf29* | 22 | -1.168 | 8.77E-05 | 0.0126 |
| *IGFBP6* | 12 | 1.383 | 8.97E-05 | 0.0128 |
| *LRRCC1* | 8 | -1.285 | 9.28E-05 | 0.0130 |
| *NOS3* | 7 | 1.179 | 9.56E-05 | 0.0132 |
| *BHLHE22* | 8 | 1.205 | 9.68E-05 | 0.0133 |
| *INVS* | 9 | 1.073 | 9.83E-05 | 0.0133 |
| *ILVBL* | 19 | -1.090 | 0.0001 | 0.0137 |
| *MTHFD2* | 2 | 1.238 | 0.0001 | 0.0138 |
| *SMAP2* | 1 | 1.161 | 0.0001 | 0.0141 |
| *ANK2* | 4 | 1.220 | 0.0001 | 0.0156 |
| *C19orf22* | 19 | 1.149 | 0.0001 | 0.0157 |
| *LOC643733* | 11 | -1.131 | 0.0001 | 0.0162 |
| *ANKRD40* | 17 | 1.072 | 0.0002 | 0.0198 |
| *GPIHBP1* | 8 | -1.323 | 0.0002 | 0.0199 |
| *SLC2A11* | 22 | 1.350 | 0.0002 | 0.0199 |
| *SRPX2* | X | 1.173 | 0.0002 | 0.0199 |
| *EVL* | 14 | -1.142 | 0.0002 | 0.0207 |
| *KRT8* | 12 | 1.232 | 0.0002 | 0.0212 |
| *C5orf13* | 5 | -1.136 | 0.0002 | 0.0212 |
| *PGC* | 6 | 1.339 | 0.0002 | 0.0212 |
| *COPZ2* | 17 | 1.115 | 0.0002 | 0.0215 |
| *DYRK3* | 1 | 1.213 | 0.0002 | 0.0215 |
| *FOLR2* | 11 | 1.234 | 0.0002 | 0.0215 |
| *HMOX1* | 22 | 1.356 | 0.0002 | 0.0215 |
| *C11orf96* | 11 | 1.321 | 0.0002 | 0.0215 |
| *SCARA5* | 8 | 1.381 | 0.0002 | 0.0215 |
| *USP9X* | X | -1.084 | 0.0002 | 0.0215 |
| *NDST1* | 5 | -1.158 | 0.0002 | 0.0218 |
| *STX19* | 3 | -1.195 | 0.0002 | 0.0218 |
| *WDFY1* | 2 | -1.101 | 0.0002 | 0.0218 |
| *LMNA* | 1 | 1.256 | 0.0002 | 0.0222 |
| *PADI4* | 1 | 1.378 | 0.0002 | 0.0222 |
| *MAP1B* | 5 | 1.234 | 0.0002 | 0.0234 |
| *TERF1* | 8 | -1.107 | 0.0002 | 0.0236 |
| *PIN4* | X | -1.114 | 0.0002 | 0.0237 |
| *SELP* | 1 | 1.191 | 0.0002 | 0.0237 |
| *CIR1* | 2 | -1.172 | 0.0002 | 0.0240 |
| *SGPL1* | 10 | 1.077 | 0.0002 | 0.0240 |
| *SAT1* | X | 1.119 | 0.0003 | 0.0242 |
| *PODN* | 1 | 1.233 | 0.0003 | 0.0251 |
| *ASRGL1* | 11 | 1.109 | 0.0003 | 0.0254 |
| *RPL13AP17* | 7 | -1.356 | 0.0003 | 0.0254 |
| *TTC31* | 2 | -1.098 | 0.0003 | 0.0254 |
| *SFRP1* | 8 | 1.359 | 0.0003 | 0.0262 |
| *RRNAD1* | 1 | -1.076 | 0.0003 | 0.0272 |
| *IRX3* | 16 | -1.304 | 0.0003 | 0.0274 |
| *DIMT1* | 5 | 1.071 | 0.0003 | 0.0279 |
| *SEPSECS* | 4 | -1.083 | 0.0003 | 0.0281 |
| *GSTA4* | 6 | -1.150 | 0.0003 | 0.0283 |
| *IFNGR2* | 21 | 1.087 | 0.0003 | 0.0294 |
| *EAF1* | 3 | 1.098 | 0.0003 | 0.0297 |
| *GIN1* | 5 | -1.090 | 0.0003 | 0.0297 |
| *PDLIM1* | 10 | 1.118 | 0.0003 | 0.0297 |
| *PPP1R14B* | 11 | 1.094 | 0.0004 | 0.0304 |
| *CXCR7* | 2 | 1.229 | 0.0004 | 0.0309 |
| *PEBP4* | 8 | -1.201 | 0.0004 | 0.0309 |
| *GKN2* | 2 | 1.203 | 0.0004 | 0.0314 |
| *MAD2L2* | 1 | 1.098 | 0.0004 | 0.0314 |
| *ANKRD50* | 4 | -1.127 | 0.0004 | 0.0314 |
| *OXR1* | 8 | -1.133 | 0.0004 | 0.0314 |
| *PTPRE* | 10 | 1.117 | 0.0004 | 0.0314 |
| *PXMP4* | 20 | -1.166 | 0.0004 | 0.0314 |
| *NEMF* | 14 | -1.138 | 0.0004 | 0.0314 |
| *TRIM24* | 7 | -1.134 | 0.0004 | 0.0314 |
| *GSTM2* | 1 | -1.120 | 0.0004 | 0.0324 |
| *ITGA5* | 12 | 1.160 | 0.0004 | 0.0324 |
| *TRIM47* | 17 | 1.141 | 0.0004 | 0.0329 |
| *PDIK1L* | 1 | -1.141 | 0.0004 | 0.0332 |
| *PRAF2* | X | 1.074 | 0.0004 | 0.0334 |
| *CHIC2* | 4 | 1.129 | 0.0005 | 0.0337 |
| *ELF4* | X | 1.101 | 0.0005 | 0.0337 |
| *GRPEL1* | 4 | 1.125 | 0.0005 | 0.0337 |
| *HBB* | 11 | 1.177 | 0.0005 | 0.0337 |
| *ALDH1A1* | 9 | -1.118 | 0.0005 | 0.0338 |
| *SRPR* | 11 | 1.080 | 0.0005 | 0.0342 |
| *C7orf36* | 7 | -1.090 | 0.0005 | 0.0347 |
| *C6orf138* | 6 | 1.046 | 0.0005 | 0.0349 |
| *SLC7A1* | 13 | 1.157 | 0.0005 | 0.0352 |
| *RBM12B* | 8 | -1.147 | 0.0005 | 0.0357 |
| *ASF1A* | 6 | -1.129 | 0.0005 | 0.0357 |
| *NFE2* | 12 | 1.181 | 0.0005 | 0.0357 |
| *OAF* | 11 | 1.214 | 0.0005 | 0.0357 |
| *ST3GAL5* | 2 | -1.108 | 0.0005 | 0.0357 |
| *TUBA4A* | 2 | 1.112 | 0.0005 | 0.0357 |
| *PHGDH* | 1 | -1.170 | 0.0005 | 0.0364 |
| *ZBED5* | 11 | -1.141 | 0.0005 | 0.0370 |
| *LOC100653336* | 2 | 1.103 | 0.0006 | 0.0377 |
| *NDRG2* | 14 | -1.138 | 0.0006 | 0.0382 |
| *ZNF704* | 8 | -1.124 | 0.0006 | 0.0392 |
| *CELF2* | 10 | 1.095 | 0.0006 | 0.0403 |
| *IL1RL1* | 2 | 1.468 | 0.0006 | 0.0403 |
| *RBM26* | 13 | -1.097 | 0.0006 | 0.0403 |
| *RDX* | 11 | -1.172 | 0.0006 | 0.0403 |
| *TTC14* | 3 | -1.168 | 0.0006 | 0.0403 |
| *AQP4* | 18 | -1.197 | 0.0006 | 0.0412 |
| *GTF2E2* | 8 | 1.058 | 0.0006 | 0.0413 |
| *KPNA1* | 3 | 1.086 | 0.0006 | 0.0414 |
| *ALDH1L2* | 12 | 1.090 | 0.0007 | 0.0416 |
| *C6orf70* | 6 | -1.069 | 0.0007 | 0.0416 |
| *LGALSL* | 2 | 1.159 | 0.0007 | 0.0416 |
| *NDEL1* | 17 | 1.130 | 0.0007 | 0.0416 |
| *FNDC4* | 2 | 1.130 | 0.0007 | 0.0418 |
| *ITGA2* | 5 | 1.212 | 0.0007 | 0.0418 |
| *TSHZ2* | 20 | 1.199 | 0.0007 | 0.0418 |
| *PSIP1* | 9 | -1.142 | 0.0007 | 0.0422 |
| *DCUN1D3* | 16 | 1.185 | 0.0007 | 0.0424 |
| *MAFB* | 20 | 1.148 | 0.0007 | 0.0424 |
| *ARHGEF6* | X | -1.089 | 0.0007 | 0.0426 |
| *CCDC3* | 10 | 1.205 | 0.0007 | 0.0426 |
| *TLR4* | 9 | 1.122 | 0.0007 | 0.0426 |
| *AXIN2* | 17 | -1.180 | 0.0007 | 0.0429 |
| *IL1R1* | 2 | 1.152 | 0.0007 | 0.0438 |
| *MBLAC2* | 5 | -1.091 | 0.0007 | 0.0438 |
| *C14orf28* | 14 | -1.138 | 0.0008 | 0.0442 |
| *ALDH6A1* | 14 | -1.144 | 0.0008 | 0.0446 |
| *CES1* | 16 | -1.225 | 0.0008 | 0.0457 |
| *CLCNKA* | 1 | -1.116 | 0.0008 | 0.0457 |
| *FJX1* | 11 | 1.205 | 0.0008 | 0.0465 |
| *SELK* | 3 | 1.114 | 0.0008 | 0.0466 |
| *GNPNAT1* | 14 | 1.067 | 0.0008 | 0.0470 |
| *LETMD1* | 12 | -1.093 | 0.0008 | 0.0470 |
| *ZNF503* | 10 | -1.200 | 0.0008 | 0.0472 |
| *AGGF1* | 5 | -1.114 | 0.0009 | 0.0473 |
| *C9orf21* | 9 | 1.116 | 0.0009 | 0.0473 |
| *DES* | 2 | 1.229 | 0.0009 | 0.0473 |
| *FKBP11* | 12 | 1.191 | 0.0009 | 0.0473 |
| *ORC3* | 6 | -1.089 | 0.0009 | 0.0473 |
| *PSMC5* | 17 | 1.056 | 0.0009 | 0.0473 |
| *SMC4* | 3 | -1.153 | 0.0009 | 0.0473 |
| *TRMT2B* | X | -1.104 | 0.0009 | 0.0473 |
| *TUBA1C* | 12 | 1.137 | 0.0009 | 0.0473 |
| *ZBTB39* | 12 | 1.049 | 0.0009 | 0.0473 |
| *PRADC1* | 2 | 1.092 | 0.0009 | 0.0475 |
| *STIM1* | 11 | 1.070 | 0.0009 | 0.0478 |
| *EFCAB7* | 1 | -1.094 | 0.0009 | 0.0479 |
| *WIPI1* | 17 | 1.084 | 0.0009 | 0.0479 |
| *CIRBP* | 19 | -1.068 | 0.0009 | 0.0493 |
| *PRKCB* | 16 | 1.134 | 0.0010 | 0.0493 |
| *C12orf29* | 12 | -1.104 | 0.0010 | 0.0497 |
