# Supplementary material for: Human Lung Tissue Transcriptome: Influence of Sex and Age
Source: PLoS One. 2016 Nov 30;11(11):e0167460. doi: 10.1371/journal.pone.0167460 (PMC5130276; doi:10.1371/journal.pone.0167460)
Supplement: S3 Table — (DOCX) [file pone.0167460.s003.docx]

**S3 Table.** List of 217 genes whose expression in lung tissue associated with age at surgery in the discovery series, presented in order of false discovery rate (FDR) values.

| **Gene symbol** | **Chromosome** | **Estimate beta** | ***P* value** | **FDR** |
| --- | --- | --- | --- | --- |
| *FRZB* | 2 | 0.0234 | 3.66E-10 | 4.06E-06 |
| *MYO1D* | 17 | 0.0097 | 1.63E-09 | 9.02E-06 |
| *ITGBL1* | 13 | 0.0183 | 3.27E-09 | 1.21E-05 |
| *LOC100653336* | 2 | 0.0112 | 1.85E-08 | 5.13E-05 |
| *SLC22A3* | 6 | -0.0189 | 3.61E-08 | 8.01E-05 |
| *WISP2* | 20 | 0.0263 | 4.88E-08 | 9.02E-05 |
| *MOXD1* | 6 | 0.0194 | 7.60E-08 | 1.20E-04 |
| *RCAN2* | 6 | 0.0154 | 1.13E-07 | 1.56E-04 |
| *ZMAT3* | 3 | 0.0166 | 1.50E-07 | 1.84E-04 |
| *SELM* | 22 | 0.0109 | 1.73E-07 | 1.92E-04 |
| *CCDC3* | 10 | 0.0194 | 5.94E-07 | 5.99E-04 |
| *TPM1* | 15 | 0.0139 | 6.62E-07 | 6.12E-04 |
| *IGFBP7* | 4 | 0.0156 | 9.24E-07 | 7.77E-04 |
| *PDE1A* | 2 | 0.0149 | 9.81E-07 | 7.77E-04 |
| *LAMP3* | 3 | -0.0134 | 1.21E-06 | 8.94E-04 |
| *FANCE* | 6 | -0.0108 | 1.63E-06 | 0.0011 |
| *PLA1A* | 3 | 0.0209 | 1.88E-06 | 0.0012 |
| *DPT* | 1 | 0.0210 | 2.63E-06 | 0.0016 |
| *CRYAB* | 11 | 0.0138 | 2.80E-06 | 0.0016 |
| *CDKN2C* | 1 | 0.0151 | 3.17E-06 | 0.0017 |
| *PPP1R14C* | 6 | -0.0142 | 3.23E-06 | 0.0017 |
| *TSPAN8* | 12 | 0.0145 | 3.28E-06 | 0.0017 |
| *RNF141* | 11 | -0.0092 | 3.97E-06 | 0.0019 |
| *DENND5B* | 12 | 0.0110 | 4.79E-06 | 0.0020 |
| *NOX4* | 11 | 0.0133 | 4.74E-06 | 0.0020 |
| *PLAG1* | 8 | -0.0106 | 4.56E-06 | 0.0020 |
| *AEBP1* | 7 | 0.0182 | 5.39E-06 | 0.0021 |
| *HK2* | 2 | -0.0139 | 5.44E-06 | 0.0021 |
| *PTGDS* | 9 | 0.0170 | 5.40E-06 | 0.0021 |
| *NECAB1* | 8 | -0.0175 | 6.67E-06 | 0.0025 |
| *KCTD5* | 16 | -0.0125 | 6.93E-06 | 0.0025 |
| *DIRAS3* | 1 | 0.0089 | 8.77E-06 | 0.0030 |
| *INPP4B* | 4 | 0.0104 | 9.37E-06 | 0.0031 |
| *OLFM1* | 9 | 0.0128 | 9.19E-06 | 0.0031 |
| *CPXM2* | 10 | 0.0203 | 9.92E-06 | 0.0031 |
| *CTLA4* | 2 | 0.0222 | 1.19E-05 | 0.0032 |
| *FKBP15* | 9 | -0.0094 | 1.20E-05 | 0.0032 |
| *MXRA8* | 1 | 0.0121 | 1.20E-05 | 0.0032 |
| *NECAB3* | 20 | -0.0089 | 1.13E-05 | 0.0032 |
| *OSBPL11* | 3 | -0.0112 | 1.18E-05 | 0.0032 |
| *PARP8* | 5 | 0.0075 | 1.13E-05 | 0.0032 |
| *PMEPA1* | 20 | 0.0112 | 1.29E-05 | 0.0034 |
| *SMC4* | 3 | -0.0133 | 1.38E-05 | 0.0036 |
| *C7* | 5 | 0.0186 | 1.49E-05 | 0.0036 |
| *ELN* | 7 | 0.0171 | 1.46E-05 | 0.0036 |
| *LAX1* | 1 | 0.0152 | 1.45E-05 | 0.0036 |
| *HEPH* | X | 0.0118 | 1.74E-05 | 0.0040 |
| *NRGN* | 11 | -0.0148 | 1.76E-05 | 0.0040 |
| *SLPI* | 20 | -0.0146 | 1.78E-05 | 0.0040 |
| *MIS18BP1* | 14 | -0.0131 | 1.92E-05 | 0.0043 |
| *CCDC88A* | 2 | -0.0102 | 2.14E-05 | 0.0047 |
| *GSTA4* | 6 | -0.0117 | 2.24E-05 | 0.0048 |
| *CPAMD8* | 19 | -0.0083 | 2.46E-05 | 0.0052 |
| *CTHRC1* | 8 | 0.0234 | 2.61E-05 | 0.0053 |
| *HSD17B6* | 12 | -0.0172 | 2.65E-05 | 0.0053 |
| *C2orf40* | 2 | 0.0204 | 3.07E-05 | 0.0061 |
| *MRPS6* | 21 | 0.0057 | 3.32E-05 | 0.0065 |
| *COX7A1* | 19 | 0.0114 | 3.39E-05 | 0.0065 |
| *RERGL* | 12 | 0.0229 | 3.58E-05 | 0.0067 |
| *AGRP* | 16 | -0.0227 | 3.74E-05 | 0.0069 |
| *CPB2* | 13 | 0.0227 | 4.05E-05 | 0.0069 |
| *CSRP2* | 12 | 0.0119 | 3.98E-05 | 0.0069 |
| *EYA4* | 6 | 0.0145 | 3.80E-05 | 0.0069 |
| *GPD1* | 12 | -0.0149 | 4.09E-05 | 0.0069 |
| *RACGAP1* | 12 | -0.0071 | 4.09E-05 | 0.0069 |
| *SMOC2* | 6 | 0.0179 | 4.11E-05 | 0.0069 |
| *C1S* | 12 | 0.0119 | 4.24E-05 | 0.0070 |
| *VTRNA1-3* | 5 | 0.0201 | 4.28E-05 | 0.0070 |
| *OSCAR* | 19 | -0.0147 | 4.84E-05 | 0.0078 |
| *RBM38* | 20 | 0.0098 | 4.97E-05 | 0.0079 |
| *NLRC4* | 2 | -0.0105 | 5.09E-05 | 0.0079 |
| *GZMK* | 5 | 0.0194 | 5.38E-05 | 0.0083 |
| *DKK3* | 11 | 0.0107 | 5.80E-05 | 0.0088 |
| *DUXA* | 19 | 0.0172 | 6.51E-05 | 0.0097 |
| *PCSK5* | 9 | 0.0110 | 6.60E-05 | 0.0097 |
| *RAB6B* | 3 | 0.0079 | 6.75E-05 | 0.0097 |
| *SELENBP1* | 1 | -0.0106 | 6.72E-05 | 0.0097 |
| *SPON1* | 11 | 0.0125 | 7.20E-05 | 0.0101 |
| *TRAM2* | 6 | 0.0091 | 7.22E-05 | 0.0101 |
| *AFF3* | 2 | -0.0122 | 7.68E-05 | 0.0103 |
| *SNORD14C* | 11 | 0.0116 | 7.62E-05 | 0.0103 |
| *4-Sep* | 17 | 0.0102 | 7.49E-05 | 0.0103 |
| *SPOCD1* | 1 | -0.0181 | 7.58E-05 | 0.0103 |
| *SVIL* | 10 | -0.0068 | 8.36E-05 | 0.0110 |
| *CTGF* | 6 | 0.0136 | 9.40E-05 | 0.0120 |
| *HABP4* | 9 | 0.0076 | 9.29E-05 | 0.0120 |
| *PRAM1* | 19 | -0.0146 | 9.50E-05 | 0.0120 |
| *SOD3* | 4 | 0.0117 | 9.38E-05 | 0.0120 |
| *ADAMTSL2* | 9 | 0.0143 | 0.0001 | 0.0130 |
| *LOC283683* | 15 | -0.0121 | 0.0001 | 0.0130 |
| *FABP5* | 8 | -0.0152 | 0.0001 | 0.0130 |
| *RBM47* | 4 | -0.0084 | 0.0001 | 0.0130 |
| *ACACA* | 17 | -0.0066 | 0.0001 | 0.0133 |
| *BNIPL* | 1 | -0.0144 | 0.0001 | 0.0133 |
| *LINC00478* | 21 | 0.0087 | 0.0001 | 0.0133 |
| *FHL2* | 2 | 0.0186 | 0.0001 | 0.0133 |
| *JPH1* | 8 | -0.0094 | 0.0001 | 0.0133 |
| *LAMA2* | 6 | 0.0127 | 0.0001 | 0.0133 |
| *SECISBP2L* | 15 | -0.0123 | 0.0001 | 0.0137 |
| *PLCD3* | 17 | 0.0060 | 0.0001 | 0.0142 |
| *SNTB1* | 8 | -0.0096 | 0.0001 | 0.0142 |
| *TMEM45A* | 3 | 0.0115 | 0.0001 | 0.0142 |
| *FMO3* | 1 | 0.0131 | 0.0001 | 0.0144 |
| *SGCA* | 17 | 0.0121 | 0.0001 | 0.0157 |
| *ABCA3* | 16 | -0.0111 | 0.0002 | 0.0163 |
| *CFTR* | 7 | -0.0130 | 0.0002 | 0.0163 |
| *COL16A1* | 1 | 0.0130 | 0.0002 | 0.0163 |
| *FZD5* | 2 | -0.0088 | 0.0002 | 0.0163 |
| *UBQLN3* | 11 | 0.0076 | 0.0002 | 0.0163 |
| *LTA4H* | 12 | -0.0099 | 0.0002 | 0.0168 |
| *PDGFD* | 11 | 0.0119 | 0.0002 | 0.0171 |
| *SRPX2* | X | 0.0114 | 0.0002 | 0.0171 |
| *FGR* | 1 | -0.0107 | 0.0002 | 0.0172 |
| *CA4* | 17 | -0.0179 | 0.0002 | 0.0177 |
| *SERPINA3* | 14 | 0.0208 | 0.0002 | 0.0180 |
| *C12orf24* | 12 | 0.0055 | 0.0002 | 0.0188 |
| *CACNA2D2* | 3 | -0.0105 | 0.0002 | 0.0188 |
| *VCAM1* | 1 | 0.0219 | 0.0002 | 0.0188 |
| *PDLIM3* | 4 | 0.0151 | 0.0002 | 0.0191 |
| *SCARF2* | 22 | 0.0097 | 0.0002 | 0.0196 |
| *AIF1L* | 9 | 0.0119 | 0.0002 | 0.0209 |
| *SSC5D* | 19 | 0.0168 | 0.0002 | 0.0209 |
| *SYNPO2* | 4 | 0.0127 | 0.0002 | 0.0214 |
| *TRNP1* | 1 | 0.0104 | 0.0002 | 0.0214 |
| *ANK3* | 10 | -0.0107 | 0.0002 | 0.0216 |
| *HSD17B4* | 5 | -0.0067 | 0.0002 | 0.0216 |
| *PION* | 7 | -0.0114 | 0.0003 | 0.0236 |
| *TESC* | 12 | 0.0096 | 0.0003 | 0.0241 |
| *RABGGTB* | 1 | -0.0066 | 0.0003 | 0.0255 |
| *SLC22A15* | 1 | -0.0098 | 0.0003 | 0.0280 |
| *HSPB6* | 19 | 0.0148 | 0.0003 | 0.0284 |
| *FABP5P3* | 7 | -0.0130 | 0.0003 | 0.0284 |
| *AGR2* | 7 | -0.0121 | 0.0004 | 0.0295 |
| *COPZ2* | 17 | 0.0075 | 0.0004 | 0.0295 |
| *CRTAC1* | 10 | -0.0138 | 0.0004 | 0.0295 |
| *DUSP14* | 17 | 0.0139 | 0.0004 | 0.0295 |
| *EYA2* | 20 | 0.0119 | 0.0004 | 0.0295 |
| *SLC39A10* | 2 | -0.0066 | 0.0004 | 0.0305 |
| *LOC388588* | 1 | -0.0090 | 0.0004 | 0.0317 |
| *FAM82A2* | 15 | -0.0088 | 0.0004 | 0.0318 |
| *NRSN2* | 20 | 0.0073 | 0.0004 | 0.0319 |
| *FSTL3* | 19 | 0.0117 | 0.0004 | 0.0321 |
| *F8* | X | 0.0137 | 0.0004 | 0.0323 |
| *SELP* | 1 | 0.0120 | 0.0004 | 0.0323 |
| *RGS12* | 4 | -0.0096 | 0.0004 | 0.0334 |
| *TMEM119* | 12 | 0.0126 | 0.0004 | 0.0334 |
| *WDSUB1* | 2 | -0.0073 | 0.0004 | 0.0334 |
| *CXCL9* | 4 | 0.0287 | 0.0004 | 0.0335 |
| *LOC401321* | 7 | -0.0079 | 0.0005 | 0.0337 |
| *CYP27A1* | 2 | -0.0131 | 0.0005 | 0.0340 |
| *CHDH* | 3 | -0.0110 | 0.0005 | 0.0346 |
| *MCOLN1* | 19 | -0.0089 | 0.0005 | 0.0346 |
| *FBLN1* | 22 | 0.0168 | 0.0005 | 0.0358 |
| *MMP23B* | 1 | 0.0102 | 0.0005 | 0.0358 |
| *SSPN* | 12 | 0.0100 | 0.0005 | 0.0359 |
| *GEM* | 8 | 0.0180 | 0.0005 | 0.0360 |
| *GPC6* | 13 | 0.0109 | 0.0005 | 0.0360 |
| *PRSS23* | 11 | 0.0108 | 0.0005 | 0.0366 |
| *ATP6AP1* | X | -0.0068 | 0.0006 | 0.0378 |
| *GAMT* | 19 | 0.0073 | 0.0006 | 0.0378 |
| *LCN2* | 9 | -0.0258 | 0.0005 | 0.0378 |
| *TAGLN* | 11 | 0.0143 | 0.0005 | 0.0378 |
| *TC2N* | 14 | -0.0092 | 0.0006 | 0.0378 |
| *ZNF275* | X | 0.0071 | 0.0006 | 0.0378 |
| *TMEM33* | 4 | -0.0070 | 0.0006 | 0.0379 |
| *SNX10* | 7 | -0.0148 | 0.0006 | 0.0384 |
| *DDX28* | 16 | -0.0074 | 0.0006 | 0.0386 |
| *TKT* | 3 | -0.0072 | 0.0006 | 0.0388 |
| *ADPGK* | 15 | -0.0055 | 0.0006 | 0.0401 |
| *CAND2* | 3 | 0.0070 | 0.0006 | 0.0402 |
| *FMNL2* | 2 | -0.0085 | 0.0006 | 0.0402 |
| *GPR98* | 5 | -0.0091 | 0.0006 | 0.0402 |
| *CERCAM* | 9 | 0.0118 | 0.0006 | 0.0402 |
| *MMP2* | 16 | 0.0087 | 0.0006 | 0.0402 |
| *RHOT1* | 17 | -0.0080 | 0.0006 | 0.0402 |
| *IL17D* | 13 | 0.0090 | 0.0006 | 0.0405 |
| *FAM150B* | 2 | 0.0205 | 0.0007 | 0.0405 |
| *TMEM176B* | 7 | 0.0094 | 0.0006 | 0.0405 |
| *ZNF518B* | 4 | -0.0063 | 0.0007 | 0.0407 |
| *ABLIM2* | 4 | 0.0072 | 0.0007 | 0.0416 |
| *EFHD1* | 2 | 0.0141 | 0.0007 | 0.0416 |
| *GLT25D1* | 19 | -0.0082 | 0.0007 | 0.0416 |
| *LTBP2* | 14 | 0.0115 | 0.0007 | 0.0416 |
| *TMEM178* | 2 | 0.0119 | 0.0007 | 0.0416 |
| *HIST1H2BK* | 6 | 0.0077 | 0.0007 | 0.0418 |
| *CYB5A* | 18 | -0.0086 | 0.0007 | 0.0419 |
| *DARC* | 1 | 0.0206 | 0.0007 | 0.0419 |
| *IRX3* | 16 | -0.0178 | 0.0007 | 0.0419 |
| *ITGB5* | 3 | 0.0071 | 0.0007 | 0.0419 |
| *PLS1* | 3 | -0.0091 | 0.0007 | 0.0419 |
| *RBM12B* | 8 | -0.0096 | 0.0007 | 0.0419 |
| *TMEM14A* | 6 | 0.0065 | 0.0007 | 0.0419 |
| *ST3GAL4* | 11 | 0.0067 | 0.0007 | 0.0419 |
| *DENND4C* | 9 | -0.0099 | 0.0007 | 0.0420 |
| *SFTPD* | 10 | -0.0055 | 0.0007 | 0.0425 |
| *HP* | 16 | -0.0309 | 0.0008 | 0.0432 |
| *SPTSSA* | 14 | -0.0072 | 0.0008 | 0.0436 |
| *MSRB3* | 12 | 0.0102 | 0.0008 | 0.0438 |
| *KLHL24* | 3 | -0.0096 | 0.0008 | 0.0451 |
| *C1orf228* | 1 | -0.0088 | 0.0008 | 0.0451 |
| *VTRNA2-1* | 5 | 0.0199 | 0.0008 | 0.0451 |
| *EPHX1* | 1 | -0.0076 | 0.0009 | 0.0475 |
| *DCXR* | 17 | -0.0069 | 0.0009 | 0.0475 |
| *CD300LF* | 17 | -0.0119 | 0.0009 | 0.0480 |
| *NOL12* | 22 | -0.0080 | 0.0009 | 0.0480 |
| *PGM5* | 9 | 0.0097 | 0.0009 | 0.0480 |
| *FCHO2* | 5 | -0.0100 | 0.0009 | 0.0482 |
| *GALNTL1* | 14 | 0.0101 | 0.0009 | 0.0483 |
| *ANTXR2* | 4 | 0.0067 | 0.0009 | 0.0485 |
| *C6orf138* | 6 | 0.0030 | 0.0009 | 0.0485 |
| *CXCL17* | 19 | -0.0091 | 0.0009 | 0.0485 |
| *ITGA9* | 3 | 0.0075 | 0.0009 | 0.0485 |
| *C10orf140* | 10 | 0.0093 | 0.0009 | 0.0485 |
| *PRCP* | 11 | 0.0065 | 0.0009 | 0.0485 |
| *FDFT1* | 8 | -0.0064 | 0.0009 | 0.0487 |
| *SLC11A1* | 2 | -0.0155 | 0.0009 | 0.0487 |
| *MGP* | 12 | 0.0084 | 0.0010 | 0.0497 |
