# Supplementary material for: Human Lung Tissue Transcriptome: Influence of Sex and Age
Source: PLoS One. 2016 Nov 30;11(11):e0167460. doi: 10.1371/journal.pone.0167460 (PMC5130276; doi:10.1371/journal.pone.0167460)
Supplement: S4 Table — Of the 25 validated sex-related genes identified in adult lung tissue, 18 were also found to be differentially expressed between males and females in fetal lung tissue (GSE68896) (DOCX) [file pone.0167460.s004.docx]

**S4 Table.** **Sex-related differentially expressed genes in both adult and fetal lung tissue.** Of the 25 validated sex-related genes identified in adult lung tissue, 18 were also found to be differentially expressed between males and females in fetal lung tissue (GSE68896)

| **Gene symbol** | **Chromosome** | **Genes validated in adults (this study)** | | | **Genes from GSE68896** | | |
| --- | --- | --- | --- | --- | --- | --- | --- |
|  |  | **Fold change (M vs F).** | ***P*-value** | **FDR** | **Fold change (M vs. F)** | ***P*-value** | **FDR** |
| RPS4Y1 | Y | 48.95 | 1.58E-253 | 1.76E-249 | 13.45 | 2.38E-308 | 5.54E-304 |
| HDHD1 | X | -1.59 | 4.75E-54 | 1.75E-50 | -1.16 | 4.10E-15 | 2.45E-12 |
| ARSD | X | -1.54 | 2.08E-53 | 5.78E-50 | -1.11 | 1.09E-10 | 5.54E-08 |
| KDM6A | X | -1.51 | 2.24E-42 | 4.96E-39 | -1.40 | 2.29E-59 | 2.67E-56 |
| CD99 | X;Y | 1.34 | 3.70E-25 | 5.85E-22 | 1.38 | 2.80E-58 | 3.11E-55 |
| ZFX | X | -1.38 | 2.27E-24 | 3.14E-21 | -1.38 | 9.03E-51 | 8.42E-48 |
| EIF1AX | X | -1.34 | 1.09E-19 | 1.34E-16 | -1.24 | 1.54E-18 | 1.06E-15 |
| EIF2S3 | X | -1.23 | 1.25E-19 | 1.39E-16 | -1.10 | 3.56E-15 | 2.18E-12 |
| RPS4X | X | -1.37 | 1.98E-12 | 1.83E-09 | -1.24 | 6.37E-54 | 6.45E-51 |
| TRAPPC2 | X | -1.17 | 2.02E-10 | 1.72E-07 | -1.18 | 2.96E-10 | 1.44E-07 |
| TXLNG | X | -1.18 | 9.91E-10 | 7.33E-07 | -1.09 | 1.18E-13 | 6.40E-11 |
| ZBED1 | X;Y | 1.11 | 5.64E-08 | 3.91E-05 | 1.07 | 7.11E-17 | 4.61E-14 |
| FUNDC1 | X | -1.11 | 1.61E-07 | 1.00E-04 | -1.08 | 5.02E-09 | 2.29E-06 |
| DDX3X | X | -1.22 | 4.12E-07 | 2.00E-04 | -1.29 | 6.02E-46 | 5.39E-43 |
| OFD1 | X | -1.18 | 1.14E-06 | 5.00E-04 | -1.21 | 1.22E-20 | 8.87E-18 |
| ASMTL | X;Y | 1.11 | 2.88E-05 | 0.0064 | 1.07 | 3.91E-09 | 1.86E-06 |
| PLCXD1 | X;Y | 1.19 | 6.55E-05 | 0.0109 | 1.11 | 1.80E-08 | 7.50E-06 |
| ALDH1A1 | 9 | -1.12 | 5.00E-04 | 0.0338 | -1.15 | 3.09E-06 | 1.11E-03 |

M vs. F, males vs. females; FDR, false discovery rate.
